# Anti-CCR4 antibody activates virus specific immune response in STLV-1 infected Japanese monkey

**DOI:** 10.1186/1742-4690-12-S1-O41

**Published:** 2015-08-28

**Authors:** Kenji Sugata, Jun-ichirou Yasunaga, Kisato Nosaka, Masao Matsuoka

**Affiliations:** 1 Laboratory of Virus Control, Institute for Virus Research, Kyoto University, Kyoto, Japan; 2 Department of Hematology, Kumamoto University School of Medicine, Kumamoto, Japan

## 

Transgenic mouse model is useful to study pathogenesis of viral genes like tax and HBZ. However, it is impossible to study the host immune responses by these transgenic mice. We used simian T-cell leukemia virus type 1 (STLV-1) infected Japanese monkeys (JMs) to analyze host immune responses after treatment by anti-CCR4 monoclonal antibody (mAb). In the previous study, we reported that anti-CCR4 mAb treatment in STLV-1 infected JMs significantly reduced CCR4 positive cells and STLV-1 proviral load (PVL). In this study, we investigated the long-term effect (48 weeks) on PVL and immune responses to STLV-1 Tax (sTax) and bZIP factor (SBZ) after the anti-CCR4 mAb treatment. STLV-1 PVL in the JMs was kept at the lower level than 0 week even 48 weeks later. To further investigate the anti-CCR4 mAb induced antiviral effect, we analyzed regulatory T (Treg) cells and sTax and SBZ specific T-cell responses. Treg cells, which were CCR4 positive, were rapidly reduced after the treatment. The number of activated Treg cells (most functional population) was severely suppressed. Furthermore, anti-CCR4 mAb treatment of infected CD4 T cells from the JMs enhanced engulfment by phagocytes, which likely enhances antigen-presentation. Treated JMs showed spontaneous activation of cytotoxic T-lymphocytes (CTLs) to sTax and SBZ. However, the immune response to other antigen was not enhanced. In consistent with these data, the enhanced T-cell responses (anti-Tax and HBZ) were also observed in anti-CCR4 mAb treated ATL patients. Taken together, anti-CCR4 mAb induces CTLs to sTax/Tax and HBZ/SBZ by two different mechanisms: reduced Treg cells and enhanced antigen presentation by antibody-dependent cell-mediated phagocytosis. These effects of anti-CCR4 mAb induce prolonged suppression of PVL, which might enable long-term control of ATL and HTLV-1 infected cells.

